# The Predatory Bacterium *Bdellovibrio bacteriovorus* Aspartyl-tRNA Synthetase Recognizes tRNA^Asn^ as a Substrate

**DOI:** 10.1371/journal.pone.0110842

**Published:** 2014-10-22

**Authors:** Ariel Alperstein, Brittany Ulrich, Denise M. Garofalo, Ruth Dreisbach, Hannah Raff, Kelly Sheppard

**Affiliations:** Chemistry Department, Skidmore College, Saratoga Springs, New York, United States of America; University of Iowa, United States of America

## Abstract

The predatory bacterium *Bdellovibrio bacteriovorus* preys on other Gram-negative bacteria and was predicted to be an asparagine auxotroph. However, despite encoding asparaginyl-tRNA synthetase and glutaminyl-tRNA synthetase, *B. bacteriovorus* also contains the amidotransferase GatCAB. *Deinococcus radiodurans*, and *Thermus thermophilus* also encode both of these aminoacyl-tRNA synthetases with GatCAB. Both also code for a second aspartyl-tRNA synthetase and use the additional aspartyl-tRNA synthetase with GatCAB to synthesize asparagine on tRNA^Asn^. Unlike those two bacteria, *B. bacteriovorus* encodes only one aspartyl-tRNA synthetase. Here we demonstrate the lone *B. bacteriovorus* aspartyl-tRNA synthetase catalyzes aspartyl-tRNA^Asn^ formation that GatCAB can then amidate to asparaginyl-tRNA^Asn^. This non-discriminating aspartyl-tRNA synthetase with GatCAB thus provides *B. bacteriovorus* a second route for Asn-tRNA^Asn^ formation with the asparagine synthesized in a tRNA-dependent manner. Thus, in contrast to a previous prediction, *B. bacteriovorus* codes for a biosynthetic route for asparagine. Analysis of bacterial genomes suggests a significant number of other bacteria may also code for both routes for Asn-tRNA^Asn^ synthesis with only a limited number encoding a second aspartyl-tRNA synthetase.

## Introduction

*Bdellovibrio bacteriovorus* HD100 preys on other Gram-negative bacteria by inserting into the host's periplasm where *B. bacteriovorus* grows and replicates, taking advantage of the nutrient rich environment of the host cell [1]. Because this predatory process kills the host, *B. bacteriovorus* is being studied as a living antibiotic for therapeutic, agriculture, and waste treatment purposes [2]–[6]. Based on its genome, the bacterium was predicted to be missing biosynthetic pathways for nine of the proteinogenic amino acids including Asn, likely making *B. bacteriovorus* protein synthesis dependent on host degradation products [7].

For translation, *B. bacteriovorus* encodes all twenty aminoacyl-tRNA synthetases (aaRSs) [7], typical of δ-proteobacteria but unlike most bacteria which usually encode 18–19 aaRSs [8], [9]. The aaRSs missing are either glutaminyl-tRNA synthetase (GlnRS) and or asparaginyl-tRNA synthetase (AsnRS) [9]. In bacteria missing an AsnRS to directly ligate Asn to tRNA^Asn^, Asn is synthesized on tRNA^Asn^ using an indirect two-step pathway by taking advantage of an aspartyl-tRNA synthetase (AspRS) with relaxed tRNA specificity, a non-discriminating AspRS (ND-AspRS) [10], [11]. The ND-AspRS forms Asp-tRNA^Asn^, which is then amidated by the amidotransferase GatCAB to Asn-tRNA^Asn^ [10], [12], [13]. GatCAB can also be used for Gln-tRNA^Gln^ formation in bacteria lacking a GlnRS, about two-thirds of all known bacteria [8], [14]–[18].

Despite coding for both AsnRS and GlnRS, *B. bacteriovorus*, like other δ-proteobacteria, also encodes GatCAB [7], [8]. In most γ-proteobacteria like *Escherichia coli* that encode AsnRS and GlnRS, GatCAB is typically absent [8]. It has been hypothesized that *B. bacteriovorus* could use GatCAB for tRNA-dependent Asn synthesis given that it lacks both asparagine synthetases (AsnA and AsnB) [19]. For *B. bacteriovorus* to use the two-step Asn-tRNA^Asn^ synthetic pathway, it also must encode a ND-AspRS despite having an AsnRS. Previously, two bacteria were known to encode GlnRS and both routes for Asn-tRNA^Asn^ formation: *Deinococcus radiodurans* and *Thermus thermophilus* [10], [12], [20]–[22]. They encode both routes by acquiring an additional AspRS from archaea. The additional AspRS serves as the ND-AspRS required for tRNA-dependent Asn synthesis [10], [12], [20]–[22].

However, *B. bacteriovorus* codes for only one AspRS [7]. We therefore predicted the lone *B. bacteriovorus* AspRS is non-discriminating in order to facilitate GatCAB synthesis of Asn on tRNA^Asn^. We demonstrate that the *B. bacteriovorus* AspRS can readily form Asp-tRNA^Asn^ as the first step in tRNA-dependent Asn biosynthesis. By analyzing bacterial genomes, we found a significant number of bacteria may also encode both routes for Asn-tRNA^Asn^ synthesis including additional species with a second AspRS. However, only a limited number of bacteria encode AsnRS, GlnRS, GatCAB, and only one AspRS but neither Asn synthetase like *B. bacteriovorus*.

## Materials and Methods

### General

Oligonucleotides were from Integrated DNA Technologies (San Diego, California). *B. bacteriovorus* HD100 genomic DNA was a gift from Dr. John Tudor (Saint Joseph's University). Samples were sequenced at the Yale DNA Analysis Facility on Science Hill (New Haven, CT). Nuclease P1 and amino acids were from Sigma-Aldrich (St. Louis, MO). Phenol, ATP, and chloroform were from Fisher Scientific (Pittsburg, PA). [α-^32^P]ATP (10 mmol/µCi) was from Perkin Elmer (Shelton, CT). Polyethylenimine (PEI)-cellulose thin layer chromatography (TLC) glass plates were from EMD Millipore (Billerica, MA). Restriction enzymes, *Escherichia coli* BL21(DE3) and NEB10β strains, OneTaq DNA Polymerase, and T4 DNA ligase were from New England Biolabs (Ipswich, MA). *E. coli* JF448 was from the Yale Coli Genetic Stock Center (New Haven, CT). *E. coli trpA*34 was a gift from the Söll Laboratory at Yale University (New Haven, CT).

### Over-production and purification aaRSs

*B. bacteriovorus aspS* (Bd3311) was cloned between the *Nde*I and *Bam*HI restriction sites in pET28a to be N-terminally His_6_-tagged. *B. bacteriovorus* AspRS was overproduced using the autoinduction method [23] and purified by nickel-affinity chromatography in the same manner as the *S. aureus* AspRS following manufacturer's protocols (Qiagen) [24]. The purified enzyme was dialyzed, concentrated, and stored as described [24]. The enzyme preparation was determined>95% pure by Coomassie-stained polyacrylamide gel [24]. The *Legionella pneumophila aspS* was chemically synthesized (Life Technologies, GeneArt) and then subcloned between the *Nde*I and *Bam*HI sites in pET28a and overproduced and purified as described for the *S. aureus* AspRS [24].

The *B. bacteriovorus asnC* (Bd1054) was chemically synthesized with optimized codons for overproduction in *E. coli* (Life Technologies GeneArt). The optimization increased the number of codons, 52% to 90%, in the GeneArt's top codon class (90–100) based on frequency of codon usage in *E. coli* [25]. The gene was then subcloned into pET28a between the *Nde*I and *Bam*HI sites to be N-terminally His_6_-tagged. AsnRS was over-produced as described previously for the *S. aureus* homolog [24] using the autoinduction method [23] and purified by nickel-affinity chromatography following manufacturer's protocols (Qiagen) with a buffer of 50 mM Tris-HCl, pH 7.6 with 10 mM MgCl_2_ and 300 mM NaCl. The purified AsnRS was dialyzed in 50 mM Tris-HCl, pH 7.6 with 10 mM MgCl_2_, 30 mM NaCl, and 50% glycerol, and then concentrated and stored as described [24].

### *In vitro* transcription, tRNA folding, and ^32^P labeling

The tRNA genes were *in vitro* transcribed and the resultant tRNA was purified by chromatography as described [22]. The tRNAs were heated to 95°C for 5 min and slowly cooled to room temperature to refold with MgCl_2_ added to a final concentration of 5 mM at 65°C. Samples were stored at −20°C and ^32^P-labeled as described previously using the *E. coli* CCA-adding enzyme [8]. *Methanothermobacter thermautotrophicus* tRNA^Gln^ was *in vitro* transcribed, purified, and folded as described previously [26] and ^32^P-labeled as described previously using the *E. coli* CCA-adding enzyme [8].

### ^32^P-based tRNA aminoacylation assay

The aminoacylation activities of the aaRSs were monitored using the established ^32^P-based assay [8], [27]–[30]. The AspRS reactions contained 50 mM HEPES-KOH, pH 7.2, 30 mM KCl, 15 mM MgCl_2_, 5 mM DTT, 4 mM L-Asp, and 4 mM ATP. The AsnRS reactions contained 50 mM HEPES-KOH, pH 7.5, 30 mM KCl, 15 mM MgCl_2_, 5 mM DTT, 4 mM L-Asn, and 4 mM ATP. For plateau aminoacylation of tRNA, reactions were carried out at 37°C with 1.0 µM ^32^P-labeled tRNA, 11.0 µM tRNA, and 3.0 µM enzyme. Steady-state kinetic studies with 5 nM AspRS were carried out at 37°C with 0.055–1.0 µM ^32^P-labeled tRNA, and 0–10.0 µM tRNA over 6 min. Steady-state kinetic studies with 5 nM AsnRS were carried out at 37°C with 0.055–1.0 µM ^32^P-labeled tRNA^Asn^, and 0–12.0 µM tRNA^Asn^ over 6 min. Reaction mixtures and enzymes were pre-incubated for 30 sec at 37°C. Reactions were started by the addition of enzyme and repeated three to four times. Time points were quenched, digested, separated by TLC, processed and analyzed as described previously [8], [24], [29], [30]. The activity of the *L. pneumophila* AspRS was measured in the presence of 0.1 µM ^32^P-labelled tRNA, 50 mM HEPES-KOH, pH 7.2, 30 mM KCl, 15 mM MgCl_2_, 5 mM DTT, 4 mM L-Asp, and 4 mM ATP over 5 minutes. Reactions were started by the addition of *L. pneumophila* AspRS to a final concentration of 10 nM at 37°C. Time points were quenched, digested, separated by TLC, processed and analyzed as described previously [8], [29].

### *E. coli trpA34 in vivo* assay

The *B. bacteriovorus aspS* was cloned into pCBS2 between the *Nde*I and *Bgl*II restriction sites (pCBS2-*Bb*-*aspS*) [31]. In a similar fashion the *L. pneumophila aspS* was subcloned into pCBS2 (pCBS2-*Lp*-*aspS*). Following transformation into *E. coli trpA*34 cells, the cultures were grown and assayed as described previously on M9 minimal media agar plates with or without Trp with minor adjustments [24]. Briefly, cultures were grown overnight at 37°C in LB in the presence of ampicillin (100 µg/ml). The overnight culture was used to inoculate 5 mL of M9 minimal media supplemented with ampicillin (100 µg/ml) and all twenty amino acids (20 µg/ml each). The cultures were then grown shaking at 37°C for four hours. The samples were adjusted to the same O.D._600_ by diluting with M9 minimal media before 5 mL of adjusted culture was spun down at 1,500×g for 5 min, and washed three times with M9 minimal media supplemented with ampicillin (100 µg/ml). After washing, the samples were resuspended in 0.2 mL of M9 minimal media supplemented with ampicillin (100 µg/ml) before spotting 2 µL of culture on M9 minimal media agar ampicillin (100 µg/ml) plates with or without L-Trp (20 µg/ml), and supplemented with the other 19 amino acids (20 µg/ml each).

### *E. coli* JF448 *in vivo* assay

The *B. bacteriovorus aspS* and *gatCAB* (operon of Bd0058, Bd0059, Bd0060) were fused into an artificial operon as described previously [31]. The artificial operon was subcloned into the pCBS2 plasmid between the *Nde*I and *Bgl*II restriction sites (pCBS2-*Bb*-*aspS*-*gatCAB*) and transformed into *E. coli* JF448 cells. The cells were grown and assayed as described previously on M9 minimal media agar plates with or without Asn [24]. Briefly, cultures were grown overnight at 37°C in LB in the presence of ampicillin (100 µg/ml). The overnight culture was used to inoculate 5 mL of M9 minimal media supplemented with ampicillin (100 µg/ml) and all twenty amino acids (20 µg/ml each). The cultures were then grown shaking at 37°C for four hours before being spun down at 1,500×g for 5 min, and washed three times with M9 minimal media supplemented with ampicillin (100 µg/ml). After washing, the samples were resuspended in 1 mL of M9 minimal media supplemented with ampicillin (100 µg/ml) and then diluted to an O.D._600_ of 0.45. The samples were then diluted 100-fold in M9 minimal media before spotting 2 µL of culture on M9 minimal media agar ampicillin (100 µg/ml) plates with or without L-Asn (20 µg/ml), and supplemented with the other 19 amino acids (20 µg/ml each).

### Bioinformatic survey of bacterial genomes

Bacterial genomes representing 547 different genera were analyzed for genes encoding AsnRS (*asnS*), GlnRS (*glnS*), GatCAB (*gatC*, *gatA*, *gatB*), AsnA (*asnA*), AsnB (*asnB*), and AspRS (*aspS*). Genes were searched either in the UniProt (http://www.uniprot.org) or KEGG: Kyoto Encyclopedia of Genes and Genomes (http://www.genome.jp/kegg/) databases. Sequences were then compared to known relevant enzymes by BLAST to validate the presence of the relevant active sites and domain architecture. The *E. coli* CFT AsnRS (AAN79540), GlnRS (AAN79239), AsnA (AAN83104), and AsnB (AAN79222), the *H. pylori* J99 GatA (AAD06348) and GatB (AAD06184), and the *Deinococcus radiodurans* discriminating AspRS (AAF10918) and ND-AspRS (AAF10623) sequences were used for the analysis. This was of particular importance to distinguish AsnA, which lacks an anticodon-binding domain, from its orthologs, AspRS and AsnRS [32]. Bacterial-type AspRS sequences were distinguished from archaeal-type AspRS sequences by the presence of a GAD insertion domain specific to bacterial AspRSs [33]. When no gene was initially identified for an enzyme, a tBLASTn search was performed with an enzyme sequence from a related organism. In addition, the tRNA^Asn^ isoacceptors of these bacteria were analyzed for the presence of a U1-A72 base pair. The tRNA isoacceptors sequences studied were either from the Genomic tRNA Database (http://lowelab.ucsc.edu/GtRNAdb/) or the KEGG database (http://www.genome.jp/kegg/). The results of the survey are detailed in Table S1.

## Results

### *In vitro* aminoacylation of tRNA^Asp^ and tRNA^Asn^

Bacterial GatCAB recognizes the U1-A72 base pair present in many bacterial tRNA^Asn^ isoacceptors [34], [35]. *B. bacteriovorus* tRNA^Asn^ has a U1-A72 base pair (Table S1) meaning if aspartylated, the tRNA could serve as a substrate for GatCAB. However, the presence of tRNA^Asn^ with a U1-A72 base pair and GatCAB does not necessarily mean *B. bacteriovorus* encodes the two-step pathway for Asn-tRNA^Asn^ formation. For example, *Lactobacillus delbruekii bulgaricus* encodes tRNA^Asn^ with a U1-A72 pair along with GatCAB (Table S1) but does not synthesize Asn on tRNA^Asn^ as it lacks a ND-AspRS and uses only AsnRS to form Asn-tRNA^Asn^ [36].

For *B. bacteriovorus* to encode the two-step pathway for Asn-tRNA^Asn^ formation, the organism must code for a ND-AspRS along with GatCAB and tRNA^Asn^ with a U1-A72 base pair. Given the presence of GatCAB in *B. bacteriovorus* despite encoding GlnRS and AsnRS, and the absence of both asparagine synthetases (AsnA and AsnB) to synthesize Asn [7], we predicted the lone *B. bacteriovorus* AspRS aspartylates tRNA^Asn^ to enable the bacterium to synthesize Asn in a tRNA-dependent manner.

**Figure 1:**
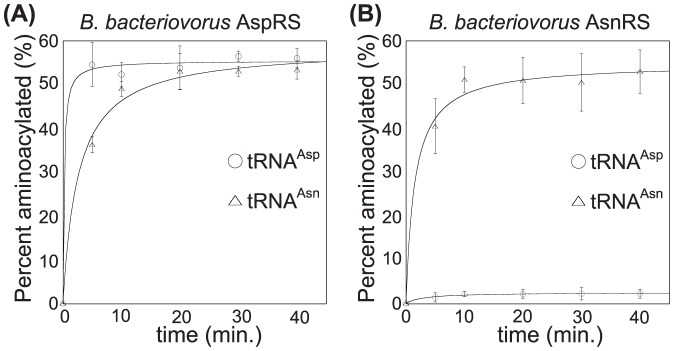
*B. bacteriovorus* AspRS aspartylates tRNA^Asn^. Aminoacylation of *in vitro* transcribed tRNA^Asp^ (○) and tRNA^Asn^ (▵) by either (A) *B. bacteriovorus* AspRS or (B) *B. bacteriovorus* AsnRS. Reactions were carried out at 37°C with 1.0 µM ^32^P-labeled tRNA^Asp or Asn^, 11.0 µM tRNA^Asp or Asn^, 4.0 mM ATP, 4.0 mM relevant amino acid (L-Asp or L-Asn) and 3.0 µM enzyme. Experiments were repeated three times and error bars represent standard deviations.

To determine if the *B. bacteriovorus* AspRS is non-discriminating, we overproduced the enzyme in *E. coli* and purified it to homogeneity [24]. The recombinant enzyme was readily able to aspartylate both tRNA^Asp^ and tRNA^Asn^ to similar levels (Figure 1A). Discriminating AspRS enzymes typically prefer tRNA^Asp^ to tRNA^Asn^ by a factor of 500–2,250 [22], [37]. In contrast and similar to other ND-AspRS enzymes [22], [24], [37], [38], the *B. bacteriovorus* AspRS preferred tRNA^Asp^ as a substrate by only 3-fold (Table 1). The difference in catalytic efficiency by AspRS was attributed to an increased *k*_cat_ with tRNA^Asp^ as a substrate (Table 1). The *B. bacteriovorus* AsnRS also readily uses tRNA^Asn^ as a substrate, reaching a similar aminoacylation plateau (Figure 1B). The tRNA^Asn^ was a better substrate for AsnRS by 3-fold with a higher *k*_cat_ compensating for an increased *K*_M_ relative to AspRS (Table 1).

**Table 1:** Aminoacylation kinetics of *B. bacteriovorus* AspRS and AsnRS at 37°C.

	*k*_cat_ (s^−1^)	*K*_M_ (µM)	*k*_cat_/*K*_M_ (s^−1^ µM^−1^)	L*
AspRS				
tRNA^Asp^	0.52±0.09	1.4±0.7	(40±20)×10^−2^	3
tRNA^Asn^	0.17±0.04	1.4±0.8	(12±7)×10^−2^	1
AsnRS				
tRNA^Asn^	0.70±0.07	2.1±0.5	(33±9)×10^−2^	3

^*^L  =  Specificity relative to the catalytic efficiency of AspRS with tRNA^Asn^ as a substrate, (*k*_cat_/*K*_M_)/(*k*_cat_/*K*_M_) of AspRS for tRNA^Asn^. Experiments were repeated three to four times and standard deviations are reported.

### *B. bacteriovorus aspS* rescues *E. coli* Trp auxotroph

**Figure 2:**
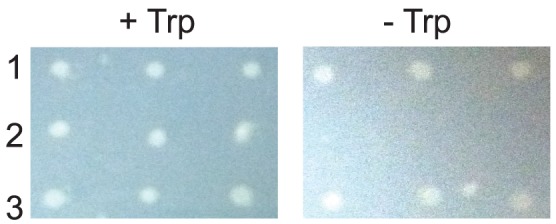
The *B. bacteriovorus aspS* rescues the Trp auxotrophy of *E. coli trpA34*. *E. coli trpA34* was grown with pCBS2 containing either 1) the *ND-aspS* from *D. radiodurans* as a positive control, 2) the *discriminating(D)-aspS* from *D. radiodurans* as a negative control, or 3) the *B. bacteriovorus aspS*. The cultures were grown in triplicate on M9 minimal media agar plates with 100 µg/ml of ampicillin in the presence (+ Trp, 20 µg/ml) or absence (- Trp) of Trp at 37°C for three days. Representative results are shown from three separate trials.

To establish whether *B. bacteriovorus* AspRS also uses tRNA^Asn^ as a substrate in a cellular context where there exists competition from other aaRSs and modified tRNA isoacceptors, we used the established *E. coli trpA*34 complementation assay [24], [31], [39]. Tryptophan synthetase alpha subunit (TrpA) is required for Trp synthesis in *E. coli*. The *trpA*34 strain is a Trp auxotroph due to mutation of codon 60 from an essential Asp codon to an Asn codon [40]. Production of a ND-AspRS in the strain rescues the phenotype, because the missense suppressor Asp-tRNA^Asn^ formed by the ND-AspRS allows decoding of the mutant Asn codon with Asp and production of active TrpA [24], [31], [39]. Consistent with our *in vitro* results demonstrating the *B. bacteriovorus* AspRS readily uses tRNA^Asn^ as a substrate, the *trpA*34 strain with the *B. bacteriovorus aspS* was able to grow in the absence of Trp (Figure 2).

### *B. bacteriovorus aspS* with *gatCAB* rescues *E. coli* Asn auxotroph

**Figure 3:**
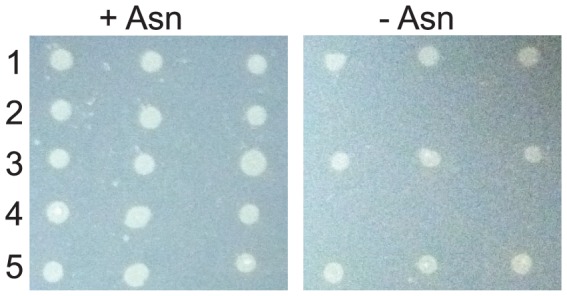
Co-production of *B. bacteriovorus* AspRS and GatCAB results in an Asn prototroph. *E. coli* JF448 was grown with pCBS2 containing either 1) the *D. radiodurans ND-aspS* and *gatCAB* or 2) the *D. radiodurans D-aspS* and *gatCAB* as positive and negative controls, respectively. 3) To control for possible toxic effects of *B. bacteriovorus aspS* expression, *E. coli* NEB10β, an Asn prototroph, was grown with pCBS2-*B. bacteriovorus aspS*. *E. coli* JF448 was also grown with pCBS2 containing either 4) the *B. bacteriovorus aspS* alone or 5) the *B. bacteriovorus aspS* and *gatCAB* in an operon together. The resultant strains were grown in triplicate on M9 minimal media agar plates with 100 µg/ml of ampicillin in the presence (+Asn, 20 µg/ml) or absence (-Asn) of Asn at 37°C for two days. Representative results are shown from three separate trials.

We predicted *B. bacteriovorus* encodes a ND-AspRS so the bacterium could synthesize Asn in a tRNA-dependent manner using GatCAB. Bacterial GatCABs readily amidate Asp-tRNA^Asn^ to Asn-tRNA^Asn^ *in vitro* [8], [12], [15]–[17], [21]. To verify co-production of *B. bacteriovorus* AspRS and GatCAB *in vivo* leads to Asn synthesis, we used the established *E. coli* JF448 system [22], [41]. The JF448 strain is an Asn auxotroph due to mutation of both Asn synthetase genes in *E. coli* [41] and the phenotype can be rescued by introducing the tRNA-dependent route for Asn biosynthesis [22], [24]. Consistent with our hypothesis, co-production of the *B. bacteriovorus* AspRS and GatCAB enabled the JF448 strain to grow in the absence of Asn in the media (Figure 3).

### Bioinformatic analysis

To determine how common it is for a bacterium to encode AsnRS, GlnRS, and GatCAB, we surveyed genomes from 547 different bacterial genera (Table S1). The three enzymes are encoded together in 68 different genera (Table 2). Like *B. bacteriovorus*, only 18 genera coded for all three while not encoding an Asn synthetase in their genomes. They represent a diverse range of bacteria from the δ-proteobacteria, the Deinococcus-Thermus, Bacteroidetes, and Verrucomicrobiae clades. All these bacteria have a tRNA^Asn^ with a U1-A72 base pair required for recognition by bacterial GatCAB, consistent with these bacteria possibly synthesizing Asn on tRNA^Asn^. A second AspRS is encoded in five of the 18 genomes, all from the Deinococcus-Thermus phylum (Tables S1 and S2). This second AspRS in this clade is of the archaeal type and may stabilize GatCAB at higher growth temperatures [42]. Similar to *B. bacteriovorus*, only 13 bacterial genera encode GlnRS, AsnRS, GatCAB and one AspRS but neither Asn synthetase (Table S1).

**Table 2:** Presence of Asn, Asn-tRNA^Asn^, and Gln-tRNA^Gln^ biosynthetic pathways in bacteria.^1^

	No AsnA/B	AsnA Only	AsnB Only	AsnA & B	Total
**GatCAB Only**	81 (81)	0 (0)	105 (105)	0 (0)	186 (186)
**AsnRS, GlnRS**	9 (8)	11 (8)	43 (35)	26 (26)	89 (77)
**GlnRS, GatCAB**	41 (41)	0 (0)	57 (57)	0 (0)	98 (98)
**AsnRS, GatCAB**	31 (28)	15 (9)	56 (55)	5 (4)	107 (96)
**AsnRS, GlnRS, GatCAB**	18 (18)	2 (2)	41 (41)	6 (6)	67 (67)

1 Representative genomes from 547 different bacterial genera were analyzed for the presence of genes coding for AsnA, AsnB, AsnRS, GlnRS, and GatCAB. The results are detailed in Table S1. In parentheses is the number of bacterial genera with a tRNA^Asn^ isoacceptor containing a U1-A72 base pair.

**Figure 4:**
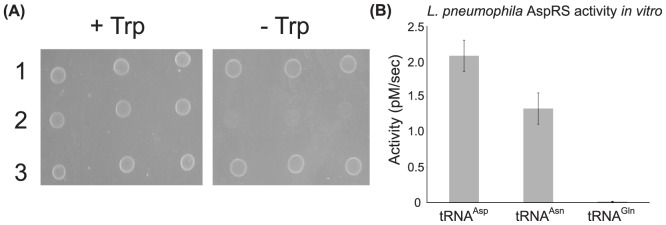
The *L. pneumophila* AspRS aspartylates tRNA^Asn^. (A) *E. coli trpA34* was grown with pCBS2 containing either 1) the *ND-aspS* from *D. radiodurans* as a positive control, 2) the *discriminating(D)-aspS* from *D. radiodurans* as a negative control, or 3) the *L. penumophila aspS*. The cultures were grown in triplicate on M9 minimal media agar plates with 100 µg/ml of ampicillin in the presence (+ Trp, 20 µg/ml) or absence (- Trp) of Trp at 37°C for three days. Representative results are shown from three separate trials. (B) Asparylation of *in vitro* transcribed tRNA^Asp^, tRNA^Asn^, and tRNA^Gln^ by the *L. pneumophila* AspRS. Reactions were carried out at 37°C with 0.1 µM ^32^P-labeled tRNA^Asp, Asn, or Gln^, 4.0 mM ATP, 4.0 mM L-Asp and 10 nM AspRS. Experiments were repeated three times and error bars represent standard deviations.

The γ-proteobacteria *L. pneumophila* belongs to the group encoding GlnRS, AsnRS, one AspRS, and GatCAB but neither Asn synthetase. The lack of an Asn synthetase suggested its AspRS recognizes tRNA^Asn^ as the first step in tRNA-dependent Asn biosynthesis as we hypothesized for *B. bacteriovorus*. We therefore tested whether the *L. pneumophila* AspRS could use tRNA^Asn^ as a substrate both *in vivo* (Fig. 4A), using the *E. coli trpA*34 assay, and *in vitro* (Fig. 4B). Like the *B. bacteriovorus* AspRS, the *L. pneumophila* was able to aspartylate tRNA^Asn^ suggesting this γ-proteobacteria potentially encodes the two-step pathway for Asn-tRNA^Asn^ formation. The *L. pneumophila* AspRS has about a 1.6-fold preference for tRNA^Asp^ over tRNA^Asn^, similar to other ND-AspRS enzymes [22], [24], [37], [38].

Beyond the Deinococcus-Thermus phylum, an additional 15 bacterial genera encode an extra AspRS (Table S2). *C. acetobutylicum* is unique in encoding three AspRSs and an additional GatCAB [18]. All 15 genomes encoded GatCAB and a tRNA^Asn^ with a U1-A72 base pair. The majority of the bacteria in this group (10 out of 15) lack an AsnRS and the indirect route with GatCAB is the only means for Asn-tRNA^Asn^ synthesis. Primarily, these bacteria are Actinobacteria in the order Actinomycetales. Of those with GatCAB and AsnRS, four are Firmicutes in the Clostridiaceae family and all encode at least one Asn synthetase. However, in the case of *C. acetobutylicum*, the AsnB is split into two halves and does not appear to be functional under normal physiological conditions with Asn synthesis being tRNA-dependent [18]. One Actinomycetales, *Amycolatopsis mediterranei* RB, also encoded an additional AspRS with AsnRS and GatCAB. It also in its genome has four *asnB* genes encoding the glutamine-dependent Asn synthetase (AsnB).

We also examined how many other bacteria potentially use both Asn-tRNA^Asn^ biosynthetic pathways. AsnRS and GatCAB are encoded in 174 bacterial genera with 163 also coding for a tRNA^Asn^ with a U1-A72 base pair (Table 2). In 46 of these bacteria, Asn synthesis could only be tRNA-dependent as they lack AsnA and AsnB, as was found in *Staphylococcus aureus* [24]. The other 117 genera encode at least one Asn synthetase. Of these, 49 also have a GlnRS suggesting GatCAB may be used for tRNA-dependent Asn synthesis including other δ-proteobacteria. *Bacteriovorax marinus* was an exception among the δ-proteobacteria as it coded for AsnRS and GlnRS but not GatCAB. It is also possible that instead the GatCAB is for Gln-tRNA^Gln^ formation but to date no bacteria are known to encode both routes for Gln-tRNA^Gln^ synthesis. The other 68 genera lack a GlnRS and retain GatCAB for Gln-tRNA^Gln^ though that does not exclude GatCAB from also being used for Asn-tRNA^Asn^ formation in these bacteria. As noted previously, AsnRS is present in all prokaryotes with AsnA, the ammonia-dependent Asn synthetase, [8], [32].

## Discussion

The *B. bacteriovorus* AspRS is non-discriminating readily able to form Asp-tRNA^Asn^. The ND-AspRS may provide the bacterium the ability synthesize Asn in a tRNA-dependent manner using GatCAB, providing a potential functional role for the amidotransferase in an organism with both AsnRS and GlnRS [19]. Thus, unlike the initial prediction after the *B. bacteriovorus* genome was sequenced [7], the bacterium does potentially encode an Asn biosynthetic pathway. Also, *B. bacteriovorus* with a ND-AspRS, GatCAB, and AsnRS could encode both routes for Asn-tRNA^Asn^ formation in addition to the direct route for Gln-tRNA^Gln^ synthesis similar to *Deinococcus radiodurans* and *Thermus thermophilus* [10], [12], [18], [20]–[22]. However, unlike those two species that acquired an additional AspRS from archaea [10], [12], [20]–[22], *B. bacteriovorus* has only one AspRS.

Acquisition of an additional AspRS in bacteria is rare. The second AspRS may provide an advantage in particular environmental niches. In the case of *T. thermophilus*, GatCAB binding to the archaeal-type ND-AspRS stabilizes the amidotransferase at elevated temperatures and the second AspRS may be an adaptation to a thermophilic environment [42]. The second AspRS in other thermophiles may also serve the same purpose in addition to allowing the bacteria to synthesize Asn in a tRNA-dependent manner. For *C. acetobutylicum*, the additional AspRS enzymes and GatCAB have been linked to the organism switching from acidogenesis to solventogenesis [18], [43].

Why *B. bacteriovorus* may still retain the tRNA-dependent route for Asn production despite coding for AsnRS is not clear. The organism is unable to synthesize eight other proteinogenic amino acids [7]. During growth away from a host cell, basal protein synthesis in *B. bacteriovorus* with those eight amino acids requires recycling them from protein degradation [7]. Recycling Asn residues may be problematic as Asn residues in polypeptides are susceptible to deamidation [44], [45]. ND-AspRS and GatCAB could provide *B. bacteriovorus* the means to compensate for deamidation of Asn residues by synthesizing Asn from Asp on tRNA^Asn^. The route would also provide direct coupling of Asn synthesis for use in translation [9]. In addition, the indirect pathway would allow *B. bacteriovorus* to convert Asp to Asn from hosts that underutilize Asn. Conversely, *B. bacteriovorus* may retain AsnRS to take advantage of the Asn provided by a host as well as to efficiently recycle non-deamidated Asn residues following protein degradation as has been hypothesized in *T. thermophilus*, *D. radiodurans*, and *S. aureus* [10], [12], [22], [24].

It is also possible one of the *B. bacteriovorus* Asn-tRNA^Asn^ pathways is non-functional. However, it should be noted *asnS* expression increased significantly during *B. bacteriovorus* growth in a host cell like the other aaRS genes including AspRS with only minimal increased expression of the GatCAB genes [46]. The increased expression of the aaRS genes during the growth phase was after initial predation by *B. bacteriovorus* as expression levels were unchanged 30 min. after host infection like the GatCAB genes [47]. Interestingly, host-independent *B. bacteriovorus* cultures grown in the presence of peptone and tryptone exhibited increases in the expression of not only *aspS* and *asnS* but also the genes for GatCAB [47]. The available gene expression results are consistent with a role for GatCAB in *B. bacteriovorus* when free-living and AsnRS during growth when Asn is present either from a host or in the media. Such a scenario would be similar to what is hypothesized in *S. aureus*, that encodes a ND-AspRS, GatCAB, and AsnRS but lacks a GlnRS and either Asn synthetase [24]. The *S. aureus* GatCAB is predicted for Gln-tRNA^Gln^ formation and tRNA-dependent Asn biosynthesis, while AsnRS is predicted for growth in Asn-rich environments like the human body [24].

Interestingly, *L. pneumophila* like *B. bacteriovorus* is capable of free-living and growth in a host cell [48]. The presence of a ND-AspRS along with GlnRS, AsnRS and GatCAB in *L. pneumophila* raises the possibility it may also encode both routes for Asn-tRNA^Asn^ formation. In *L. pneumophila*, the genes for AsnRS, ND-AspRS, and GatCAB are all up regulated during post-exponential and transmissive growth phases, suggesting a role in the organism's life cycle [49].

Two distinct routes for Asn-tRNA^Asn^ formation in bacteria like *B. bacteriovorus* were likely acquired in a stepwise manner. Both AspRS and GatCAB were likely present in the last universal common ancestor (LUCA) while AsnRS evolved from a duplication of an archaeal AspRS [32], [50], [51]. Therefore, it has been hypothesized that Asn-tRNA^Asn^ formation in LUCA was via the indirect pathway using a ND-AspRS and GatCAB [50], [51]. Under this scenario, early bacteria likely also used a ND-AspRS and GatCAB for Asn-tRNA^Asn^ formation [51]. Consistent with that hypothesis, the phylogenies of the GatCAB subunits and bacterial-type AspRSs suggest they were vertically inherited in bacteria [51], [52]. AsnRS was later likely acquired in different bacterial lineages via horizontal gene transfer from archaea [32], [50], [52].

In some bacterial species like *L. delbruekii bulgaricus*, the presence of AsnRS to aminoacylate tRNA^Asn^ appears to have lessened the selective pressure for AspRS to recognize tRNA^Asn^ as a substrate, facilitating the AspRS to evolve specicity for just tRNA^Asp^ [36]. Accordingly, the role of the *L. delbruekii bulgaricus* GatCAB is for only Gln-tRNA^Gln^ synthesis [36]. In *B. bacteriovorus*, AspRS retained its relaxed tRNA specificity along with GatCAB after acquisition of AsnRS possibly for tRNA-dependent Asn biosynthesis as was found in *S. aureus* [24]. Given the above and the presence of GatCAB and AsnRS in most δ-proteobacteria, the ancestral δ-proteobacteria likely encoded GatCAB for Asn-tRNA^Asn^ formation before acquiring AsnRS to directly attach the Asn to tRNA^Asn^. As many δ-proteobacteria, like the predatory bacterium *Myxococcus xanthus* [53] and the metal-reducing anaerobic *Geobacter metallireducens* [54], encode one or more asparagine synthetase (AsnA and or AsnB) along with AsnRS and GlnRS, the retention of GatCAB in these bacteria maybe vestigial. However, it may be beneficial for these bacteria to retain both routes for Asn-tRNA^Asn^ formation as hypothesized for *B. bacteriovorus*, *L. pneumophila*, and *S. aureus* [24].

It is unclear how many other bacteria encode both routes for Asn-tRNA^Asn^ formation. Based on our genomic analysis, about 30% of bacterial genera surveyed could code for both routes as they encode GatCAB, AsnRS, and tRNA^Asn^ isoacceptors with a U1-A72 base pair. In the few bacteria like *B. bacteriovorus* that also encode GlnRS but neither Asn synthetase, it is likely GatCAB was retained for tRNA-dependent Asn production. Similarly, those bacteria like *S. aureus* without a GlnRS and both Asn synthetases may also use GatCAB for tRNA-dependent Asn biosynthesis in addition to Gln-tRNA^Gln^ formation [24]. However, the majority of bacteria that encode AsnRS and GatCAB also code for at least one Asn synthetase (AsnA and/or AsnB); in total, 21% of all bacterial genera analyzed. Since these organisms have alternate means to synthesize Asn, GatCAB may not be for Asn-tRNA^Asn^ formation as their AspRS enzymes might be specific for tRNA^Asp^. For example, *L. delbruekii bulgaricus* AspRS does not aspartylate tRNA^Asn^ and uses GatCAB only for Gln-tRNA^Gln^ formation [36]. Clarifying the tRNA specificity of the AspRSs in other bacteria encoding both AsnRS and GatCAB would establish how many other bacteria code for both routes for Asn-tRNA^Asn^ formation, providing the foundation to better understand how Asn metabolism and protein synthesis are integrated into bacterial physiology and adaptation to certain environmental niches.

## Supporting Information

Table S1 **Genomic analysis of bacterial genomes from 547 different genera for genes related to Asn-tRNA^Asn^, Gln-tRNA^Gln^, and Asn synthesis.**
(XLSX)

Table S2 **Genomic analysis of bacterial genomes encoding at least two AspRS enzymes for genes related to Asn-tRNA^Asn^, Gln-tRNA^Gln^, and Asn synthesis.**
(XLSX)
